# Draft Genome Sequence of a Moderately Halophilic *Bacillus megaterium* Strain, MSP20.1, Isolated from a Saltern of the Little Rann of Kutch, India

**DOI:** 10.1128/genomeA.01134-13

**Published:** 2014-01-09

**Authors:** Kamal Krishna Pal, Rinku Dey, Dharmesh Sherathia, Sejal Vanpariya, Ilaxi Patel, Trupti Dalsania, Kinjal Savsani, Bhoomika Sukhadiya, Mona Mandaliya, Manesh Thomas, Sucheta Ghorai, Rupal Rupapara, Priya Rawal, Abhi Shah, Sharmila Bhayani

**Affiliations:** Microbiology Section, Directorate of Groundnut Research, Junagadh, Gujarat, India

## Abstract

The 4.37-Mbp draft genome of a moderately halophilic *Bacillus megaterium* strain, MSP20.1, isolated from a saltern of the Little Rann of Kutch, India, is reported here. To understand the mechanism(s) of moderate halophilism and to isolate the gene(s) involved in osmotolerance and adaptation, the genome of MSP20.1 was sequenced.

## GENOME ANNOUNCEMENT

The genomes of a number of species of *Bacillus* which can thrive in the salinity gradients (0 to 35% NaCl) of the hypersaline habitats of the Great and Little Rann of Kutch, India, were sequenced recently with a view to understanding the mechanism(s) of osmotolerance (1–5). *Bacillus megaterium* strain MSP20.1 (16S rRNA GenBank accession number JF802191), isolated from a saltern of the Little Rann of Kutch, India, grows optimally at a concentration of 7.5% NaCl (range, 0 to 20%) in the growth medium, at pH 7.0 and 37°C. The genome of MSP20.1 was sequenced to gain understanding of its moderate halophilism.

The genome of MSP20.1 was sequenced using the Illumina HiSeq 2000 platform at Macrogen, Inc., South Korea, through Sequencher Tech Pvt., Ltd., Ahmedabad, India. Originally, a total of 69,373,018 paired-end reads with lengths of 72 nucleotides of 7,006,674,818 bases were generated, which, after filtering and removal of PCR duplicates, resulted in 61,462,320 reads of 6,207,694,320 bases.

The genome was assembled, *de novo*, by use of SOAPdenovo assembler v 1.05 (6). The long assembly and the order of the contigs were generated by OSLay (7), with genome coverage of about 1,200×. The draft assembly of MSP20.1 (G+C content of 36.47%) resulted in an approximate genome size of 4,363,086 bases. The draft genome consists of 78 scaffolds of 3,658,318 bases, with the remaining bases included as unplaced scaffolds, with an average scaffold size of 46,901 bp (minimum, 1,057 bp; maximum, 357,224 bp) and *N*_50_ scaffold lengths of 65,811 bp. The assembly consists of 416 contigs (292 with lengths >500 bp), 360 being in the scaffolds with an average contig length of 10,488 bp (maximum, 95,529 bp; minimum, 55 bp) and *N*_*50*_ contig lengths of 24,205 bp. All the assembly data were deposited in the DDBJ/EMBL/GenBank nucleotide sequence database.

The draft genome of MSP20.1 was annotated using the RAST server (8), Glimmer 3 (9, 10), Genemark (11), tRNAScan-SE (12), and RNAmmer (13) for predicting subsystems, coding sequences (CDS), tRNAs, and rRNA genes, etc.

Using the different software tools, we identified 4,255 CDS in the draft genome of MSP20.1, with 66 RNA genes (64 tRNA and 2 rRNA genes) and 433 subsystems. Among the CDS, 2,474 are not in a subsystem (1,050 nonhypothetical, 1,424 hypothetical), whereas 1,781 CDS (nonhypothetical, 1,692; hypothetical, 89) are in a subsystem. RAST annotation also revealed the association of 110 genes in stress responses: 26 in osmotic stress (2 in osmoregulation, 20 in choline and betaine uptake and betaine biosynthesis, and 4 in ectoine biosynthesis and regulation), 46 in oxidative stress (11 in protection from reactive oxygen species [ROS], 24 in oxidative stress, 2 in glutathione:nonredox reactions, 6 in redox-dependent regulation, 2 in glutaredoxins, and 1 in the CoA disulfide thiol-disulfide redox system), 2 in cold shock, 14 in heat shock, 1 in detoxification, and 21 in no subcategory. In addition, 410 genes have been mapped to different pathways involved in the biosynthesis and degradation of amino acids and derivatives, including 105 in branched-chain amino acid pathways and 32 in the serine-glyoxylate cycle.

Further studies are under way to understand the mechanism(s) of the moderate halophilism of *Bacillus megaterium* strain MSP20.1 and its survival in salty settings.

### Nucleotide sequence accession numbers.

This whole-genome shotgun project has been deposited at DDBJ/EMBL/GenBank under the accession number AVBB00000000. The version described in this paper is version AVBB01000000.
